# Short-term fructose feeding alters tissue metabolic pathways by modulating microRNAs expression both in young and adult rats

**DOI:** 10.3389/fcell.2023.1101844

**Published:** 2023-02-16

**Authors:** Giuseppe Petito, Antonia Giacco, Federica Cioffi, Arianna Mazzoli, Nunzia Magnacca, Susanna Iossa, Fernando Goglia, Rosalba Senese, Antonia Lanni

**Affiliations:** ^1^ Department of Environmental, Biological and Pharmaceutical Sciences and Technologies, University of Campania “L. Vanvitelli”, Caserta, Italy; ^2^ Department of Sciences and Technologies, University of Sannio, Benevento, Italy; ^3^ Department of Biology, University of Naples Federico II, Naples, Italy

**Keywords:** miRNA, fructose, insulin signaling, lipid metabolism, *de novo* lipogenesis

## Abstract

Dietary high fructose (HFrD) is known as a metabolic disruptor contributing to the development of obesity, diabetes, and dyslipidemia. Children are more sensitive to sugar than adults due to the distinct metabolic profile, therefore it is especially relevant to study the metabolic alterations induced by HFrD and the mechanisms underlying such changes in animal models of different ages. Emerging research suggests the fundamental role of epigenetic factors such as microRNAs (miRNAs) in metabolic tissue injury. In this perspective, the aim of the present study was to investigate the involvement of miR-122-5p, miR-34a-5p, and miR-125b-5p examining the effects induced by fructose overconsumption and to evaluate whether a differential miRNA regulation exists between young and adult animals. We used young rats (30 days) and adult rats (90 days) fed on HFrD for a short period (2 weeks) as animal models. The results indicate that both young and adult rats fed on HFrD exhibit an increase in systemic oxidative stress, the establishment of an inflammatory state, and metabolic perturbations involving the relevant miRNAs and their axes. In the skeletal muscle of adult rats, HFrD impair insulin sensitivity and triglyceride accumulation affecting the miR-122-5p/PTP1B/P-IRS-1(Tyr612) axis. In liver and skeletal muscle, HFrD acts on miR-34a-5p/SIRT-1: AMPK pathway resulting in a decrease of fat oxidation and an increase in fat synthesis. In addition, liver and skeletal muscle of young and adult rats exhibit an imbalance in antioxidant enzyme. Finally, HFrD modulates miR-125b-5p expression levels in liver and white adipose tissue determining modifications in *de novo* lipogenesis. Therefore, miRNA modulation displays a specific tissue trend indicative of a regulatory network that contributes in targeting genes of various pathways, subsequently yielding extensive effects on cell metabolism.

## 1 Introduction

Dietary habits have changed worldwide in recent years with a significant increase of fructose intake derived from the consumption of foods with added sugar (Lelis et al., 2020). Numerous studies have provided compelling evidence that excessive use of fructose may cause harmful metabolic events by altering the molecular signaling of tissues (Bray and Popkin, 2014). It has also been suggested that fructose exerts adverse health effects that are similar to consequences of irregular alcohol consumption (Lustig, 2013). Indeed, excessive fructose consumption will adversely affect the liver (Heinz et al., 1968; Tappy and Le, 2010), but other tissues may also endure metabolic impairment, as a consequence of its overconsumption. Large quantities of fructose may be transported out of the liver to enter the systemic circulation thus affecting other metabolically active tissues, such as adipose tissue, kidney, skeletal muscle, testis, and brain (Jones et al., 2011; Francey et al., 2019; Helsley et al., 2020). The fructose-fed rat is an animal model used to study diet-induced metabolic disorders (Tran et al., 2009) and numerous metabolic syndrome features have been observed in rats fed a high fructose diet, including insulin resistance, hyperinsulinemia, hypertriglyceridemia, and hypertension. The adverse effects of fructose feeding are dependent on the amount and duration of fructose consumption (Tran et al., 2009). Adult rats subjected to long-term fructose feeding develop hepatic steatosis (Crescenzo et al., 2013b; DiStefano, 2020), insulin resistance (Crescenzo et al., 2013a), diabetes (Moore and Fielding, 2016), and obesity (Tappy and Le, 2010; Crescenzo et al., 2014) as well as a diminished capacity to repair of mitochondrial DNA damage (Cioffi et al., 2017). However, investigations have also reported unfavorable metabolic outcomes following short-term fructose consumption (Busserolles et al., 2002; Castro et al., 2012; Castro et al., 2015; Cigliano et al., 2018). In addition, most studies on fructose overconsumption have been primarily conducted on adults, albeit children and adolescents being the main consumers of high-fructose foods and sugary beverages. Recently, Crescenzo et al. (2018) showed that after a short period of fructose intake, whole body insulin sensitivity decreased both in young and adult rats, suggesting that fructose-induced insulin resistance affected specific organs with varying severity and onset in rats of different ages. In addition, Mazzoli et al. (2022) recently demonstrated that 3 weeks of fructose overconsumption in young rats caused persistent physiological modifications in metabolically relevant tissues with no reverse response following administration of a control diet.

Since young and adults differ widely in their metabolic and physiological profiles, it is particularly important to understand the metabolic alterations induced by fructose-rich diet in specific organs and the underlying mechanisms of these alterations in animal models of different ages.

Emerging research suggests the fundamental role of epigenetic factors such as microRNAs (miRNAs) in metabolic tissue injury. MiRNAs are small, non-coding, single-stranded, endogenous molecules that are influential in regulating post-transcriptional gene expression by interacting with the 3′ untranslated region (3′UTR) of its target messenger RNA (mRNA) (Vishnoi and Rani, 2017). Increasing evidence suggests that circulating miRNAs are significantly involved in intercellular communication. Such miRNAs are detected in extracellular environments as in biological fluids, and may be classified as extracellular miRNAs. In addition, these miRNAs play an important role in the crosstalk between cells and tissues with subsequent impact on health conditions (Yamada et al., 2013; Chai et al., 2017; Mori et al., 2019). Some miRNAs are involved in the control of metabolic homeostasis and many studies have demonstrated a misregulation of the microRNA network in specific metabolic organs in insulin resistance states, obesity and Non-Alcoholic Fatty Liver Disease (NAFLD) (Chartoumpekis et al., 2012; Sud et al., 2017; Hanouskova et al., 2019; Hochreuter et al., 2022; Macvanin et al., 2022). Despite extensive research on fructose and its implications regarding tissue metabolism, few investigations have addressed the specific roles and molecular mechanisms involved in the association of miRNAs and fructose-induced metabolic alteration.

Herein, we investigate whether miR-122-5p, miR-34a-5p, and miR-125b-5p are related to the impairment of insulin signaling and lipid metabolism, examining modulation of their expression and downstream targets in various tissues of fructose-fed rats. In particular, we determine 1) the miR-122-5p/PTP1B/P-IRS(Tyr612) axis, in which protein tyrosine phosphatase 1B (PTP1B) acts as a negative regulator of insulin signaling cascade and as a direct target of miR-122-5p (Goldstein, 1993; Yang et al., 2012); 2) the miR-34a-5p/SIRT-1: AMPK pathway involved in lipid metabolism *via* Sirtuin-1 (SIRT-1), a direct target of miR-34a-5p (Yamakuchi et al., 2008; Rezk et al., 2021) and AMP-activated protein kinase (AMPK), a master regulator of cellular energy balance; and 3) the miR-125b-5p and its targets, such as the Sterol regulatory element binding protein 1 (SREBP1c) and Stearoyl-CoA desaturase-1 (SCD1), that are essential to *de novo* lipogenesis (Cheng et al., 2016; Sud et al., 2017). The aforementioned miRNAs and related pathways were selected due to their specific expression patterns and functions associated with obesity and insulin resistance in human and animal studies (Li et al., 2011; Yang et al., 2012; Zhu and Leung, 2015; Cai et al., 2020). In addition, the goal of the present study is to evaluate the early metabolic disarrangements and events that take place in young and adult animals fed on with fructose and to verify the possibility that miRNAs could mediate crosstalk between cells and tissues. Accordingly, young rats (30 days) and adult rats (90 days) fed a fructose-rich diet for a short time period (2 weeks) were used as animal models, analyzing metabolically relevant peripheral organs such as the liver, skeletal muscle, and white adipose tissue.

## 2 Materials and methods

### 2.1 Materials

General reagents were of the highest available grade and were obtained from Sigma Chemical (St. Louis, MO, United States). Precision Plus Protein™ All Blue Prestained Protein Standards were obtained from BioRad (Hercules, CA, United States). Anti-PTP1B, anti-Total OXPHOS complexes cocktail, anti-SIRT-1, anti-Mfn2, anti-GPX4, and anti-PRDX3 antibodies were acquired from Abcam (Cambridge, CA, UK). Anti-P-IRS1 (Ser307), anti-P-AMPKα (Thr172), anti-AMPK-Tot, anti-IRS1, and anti-DRP1 antibodies were obtained from Cell Signaling (Danvers, MA, United States). Anti-P-IRS1(Tyr612) antibody was obtained from Invitrogen (Carlsbad, CA, United States). Anti-FAS, anti-SPOT14, anti-SREBP1c and anti-SOD-2 antibodies were acquired from Santa Cruz Biotechnology (Dallas, TX, United States). Anti-Catalase antibody was obtained from Merck (Darmastadt, DE). Anti-GPX1 antibody was acquired from GeneTex (Irvine, CA, United States). Anti-B-ACTIN antibody was obtained from Bioss Antibodies (Woburn, MA, United States). Secondary antibodies, peroxidase anti-rabbit IgG and peroxidase anti-mouse IgG were obtained from Vector Laboratories (Burlingame, CA, United States). To detect IL-1B, TGF-B, TNF-A, and IL-6 serum levels we used the Enzyme-Linked Immunosorbent Assay-ELISA kits obtained from Invitrogen (Carlsbad, CA, United States). To detect Adiponectin serum levels we used an Enzyme-Linked Immunosorbent Assay-ELISA kits, acquired from Abcam (Cambridge, CA, UK). To detect 8-OHdG we used a DNA/RNA Oxidative Damage ELISA kit obtained from Cayman Chemical Company (Ann Arbor, MI, United States). QIAzol lysis buffer and miRNeasy micro kit were obtained from Qiagen (Hilden, Germany). To synthesize cDNA strands from RNA we used the SuperScript IV reverse Transcriptase for RT-PCR obtained from Invitrogen (Carlsbad, CA, United States). IQ SYBR Green supermix was obtained from Bio-Rad (Hercules, CA, United States). TaqMan^®^ miRNA assays for miR-122-5p, miR-34a-5p, miR-125b-5p, and RNU6B were acquired from Applied Biosystems (Foster City, CA, United States).

### 2.2 Animals and treatments

Male Sprague-Dawley rats (*Rattus norvegicus*) (Charles River, Italy), of 30 (young) or 90 (adult) days were housed one per cage in a temperature-controlled room at 23°C ± 1°C under a 12 h dark/light cycle in groups of five to six with unlimited access to water. All animals received humane care according to the criteria reported in the Guide for the Care and Use of Laboratory Animals prepared by the National Academy of Sciences and published by the National Institutes of Health. All experimental procedures involving animals were approved by the “Comitato Etico-Scientifico per la Sperimentazione Animale” of the University of Naples Federico II and authorized by the Italian Ministry of Health (260/2015-PR). Every effort was made to minimize animal pain and suffering. Seven days post acclimatization, young and adult rats were divided into four groups of six and treated as follows:

• First group (CY): young rats received a standard diet for 2 weeks;
• Second group (FY): young rats received fructose rich-diet for 2 weeks;
• Third group (CA): adult rats received a standard diet for 2 weeks;
• Fourth group (FA): adult rats received fructose rich-diet for 2 weeks.

Details of the two diets are displayed in Table 1. Each group included six animals. The minimum sample size (*n* = 6) was calculated using a G* Power Test, developed by the University of Dusseldorf (http://www.gpower.hhu.de/). The power was 0.90, effect size (f) 1.2249, and the *α* was set at 0.05. On treatment completion, the rats were anesthetized using an intraperitoneal injection of Tiopental sodico (40 mg 100g-1 BW) and decapitated. Blood was collected and centrifuged at 2,000 × g to collect plasma serum, which was divided into aliquots and stored at −20°C. The liver, skeletal muscle and visceral white adipose tissue (vWAT) were excised, weighed and immediately frozen in liquid nitrogen and stored at −80°C for subsequent processing.

**TABLE 1:** Diet composition.

	Control diet	Fructose diet
Component		
Standard chow^a^	50.5	50.5
Sunflower oil	1.5	1.5
Casein	9.2	9.2
Alphacel	9.8	9.8
Starch	20.4	—
Fructose	—	20.4
Water	6.4	6.4
AIN-76 mineral mix	1.6	1.6
AIN-76 vitamin mix	0.4	0.4
Choline	0.1	0.1
Methionine	0.1	0.1
Gross energy density, kJ/g	17.2	17.2
Metabolisable energy density, kJ/g^b^	11.1	11.1
Protein,% metabolisable energy	29.0	29.0
Lipids,% metabolisable energy	10.6	10.6
Carbohydrates,% metabolisable energy	60.4	60.4
Of which: Fructose	—	30.0
Starch	52.8	22.8
Sugars	7.6	7.6

^a^ Mucedola 4RF21; Italy.

^b^ Estimated by computation using values (kJ/g) for energy content as follows: Protein 16.736, lipid 37.656, and carbohydrate 16.736.

### 2.3 Glucose tolerance test

The Glucose tolerance test was performed the day before euthanasia. To this end, rats were fasted for 5 h from 08.00 a.m. A basal, post-absorptive blood sample was obtained from a small tail clip and placed in EDTA–coated tubes and then glucose (2 g/kg body weight) was injected intraperitoneally. Blood samples were collected after 20, 40, 60, 80, 100, and 120 min and placed in EDTA-coated tubes. The blood samples were centrifuged at 2,000 × g for 15 min at 4°C. Plasma glucose concentration was measured using the colorimetric enzymatic method (Pokler Italia, Genova, Italy).

### 2.4 Immunoassay for 8-OHdG

A competitive ELISA for 8-OHdG was performed using a DNA/RNA Oxidative Damage ELISA kit (Cayman Chemical Company, Ann Arbor, Michigan, United States) according to the manufacturer’s protocol. Serum samples were analyzed in duplicate. Standard 8-OHdG was assayed over a concentration range of 10.3–3,000 pg/mL in duplicate for each experiment.

### 2.5 Determination of triglycerides, IL-1B, TGF-B, TNF-A, IL-6, and adiponectin serum levels

Serum triglycerides were measured *via* colorimetric enzymatic method using commercial kit (SGM Italia, RM, Italy). IL-1B, TGF-B, TNF-α, and IL-6 serum levels were measured using an Enzyme-Linked Immunosorbent Assay-ELISA kit, obtained from Invitrogen (Carlsbad, CA, United States). Adiponectin serum levels were determined employing an Enzyme-Linked Immunosorbent Assay- ELISA kit obtained from Abcam (Cambridge, CA, UK).

### 2.6 Cytochrome oxidase activity on liver and skeletal muscle homogenates

To detect Cytochrome oxidase activity, fragments of liver and skeletal muscle (20 mg) were immersed in ice-cold isolation buffer (100 mM KCl, 50 mM Hepes, 5 mM MgCl2, 1 mM EDTA, 5 mM EGTA, 1 mM ATP pH 7.0.), and then homogenized in a Potter-Elvehjem homogenizer (Heidiolph Instruments, Germany). Aliquots of homogenates were then incubated for 30 min at 0°C after the addition of 1.5 mg/mL lubrol. Samples of tissue homogenates were transferred into calibrated Oxygraph-2 k (O2k, OROBOROS INSTRUMENTS, Innsbruck, Austria) 2 ml-chambers. Measurement of COX activity was performed at 37°C in a medium containing 75 mM Hepes, 30 µM cytochrome c, 10 mM malonate, 4 µM rotenone, 0.5 mM dinitrophenol, 4 mM ascorbate and 0.3 mM N,N,N′,N′-tetramethyl-P-phenylenediamine.

### 2.7 Preparation of total lysates

Tissue samples of liver, skeletal muscle and vWAT were homogenized in Lysis Buffer containing 20 mM Tris-HCl (pH 7.5), 150 mM NaCl, 1 mM EDTA, 1 mM EGTA, 2.5 mM Na2H2P2O7, 1 mM b-CH3H7O6PNa2, 1 mM Na3VO4, 1 mM PMSF, 1 mg/mL leupeptin, and 1% (v/v) Triton X-100 (Sigma-Aldrich, St. Louis, MO, United Statesa) using an UltraTurrax homogenizer, and then centrifuged at 16.000 × g in a Beckman Optima TLX Ultracentrifuge (Beckman Coulter S.p.A., Milan, Italy) for 15 min at 4°C. The supernatants were then ultra-centrifuged at 40.000× RPM in a Beckman Optima TLX ultracentrifuge for 20 min at 4°C. The protein concentrations of the supernatants of the centrifuged lysates were determined using the Bio Rad’s DC method (Bio Rad Laboratories, s.r.l., Segrate, Italy).

### 2.8 Western blot analysis

Total lysates containing 30 μg protein for liver, skeletal muscle and vWAT were loaded in each lane and were electrophoresed on SDS-PAGE gels and transferred to nitrocellulose membrane and membranes were blocked with 5% (w/v) nonfat dry milk (in TBS-T). Primary antibodies were diluted in TBS with 0.01% (v/v) Tween 20 (TBS-T) and 5% (w/v) bovine serum albumin (BSA), while secondary antibodies were diluted in TBS with 0.01% (v/v) Tween 20 (TBS-T) and 5% (w/v) nonfat dry milk. Membranes were probed with the following antibodies: polyclonal anti PTP1B (Abcam- 1:750 dilution), monoclonal anti-Total OXPHOS (Abcam- 1:500 dilution), polyclonal anti SIRT-1 (Abcam- 1:1,000 dilution), polyclonal anti P-AMPKα (Thr172) (Cell Signaling- 1:1,000 dilution), polyclonal anti AMPKα (Cell Signaling- 1:1,000 dilution), monoclonal anti IRS1 (Cell Signaling- 1:500 dilution), monoclonal anti P-IRS1 (Tyr612) (Invitrogen- 1:1,000 dilution), monoclonal anti P-IRS1 (Ser307) (Cell Signaling- 1:1,000 dilution), monoclonal anti FAS (Santa Cruz Biotechnology- 1:1,000 dilution), monoclonal anti SREBP1c (Santa Cruz Biotechnology- 1:1,000 dilution), monoclonal anti SPOT14 (Santa Cruz Biotechnology- 1:1,000 dilution), polyclonal anti PGC1α (Millipore- 1:1,000 dilution), monoclonal anti Mfn2 (Abcam- 1:1,000 dilution), monoclonal anti DRP1 (Cell Signaling- 1:1,000 dilution), monoclonal anti Catalase (Merck- 1:750 dilution), monoclonal anti SOD-2 (Santa Cruz Biotechnology- 1:500 dilution), polyclonal anti GPX1 (GeneTex- 1:1,000 dilution), monoclonal anti GPX4 (Abcam- 1:1,000 dilution), polyclonal anti PRDX-3 (Abcam- 1:1,000 dilution) and monoclonal anti B-ACTIN antibody (Bioss Antibodies- 1:1,000 dilution). As secondary antibodies we used peroxidase anti rabbit IgG (Vector Laboratories- 1:2,000–1:4,000–1:10,000 dilution) and peroxidase anti-mouse IgG (Vector Laboratories- 1:4,000 dilution). Horseradish peroxidase-conjugated secondary antibodies were used for signal detection by enhanced chemiluminescence using the Chemi Doc system and related software (Hercules, CA, United States of America).

### 2.9 miRNA isolation from serum and RT-qPCR

Total RNA, including small RNAs, were isolated from 100 uL of serum using QIAzol extraction method followed by column purification with a miRNeasy Mini kit (QIAGEN) in accordance with the manufacturer’s protocol. 400 µL of QIAzol and 80 µL of chloroform were added to 100 µL of serum, followed by centrifugation for 15 min at 12.000 × g at 4°C. 300 µL of the RNA containing aqueous phase was transferred into a new tube. RNA was precipitated with 450 uL of 100% (v/v) ethanol and loaded on miRNeasy purification columns. 700 µL of the sample was pipetted into an RNeasy MinElute spin column and centrifuged for 15 s at ≥8,000 × g at room temperature. 700 µL of Buffer RWT was added to the column and centrifuge for 15 s at ≥ 8,000 × g to rinse the column and discard the flow-through. 500 µl of buffer RPE was into columns and centrifuged for 15 s at ≥ 8,000 × g. The flow-through was discarded. Following the centrifugation, the RNeasy MinElute spin columns were placed into a new tube and were centrifuged for 5 min at ≥8,000 × g. Purified RNA was eluted from the column matrix with 20 µL of RNase free water. miRNA and total RNA yield was quantified using a Nanodrop1000 device (Thermofisher, United States). miR-122-5p and miR-34a-5p were quantified along with RNU6B (reference transcript) by RT-qPCR with TaqMan^®^ miRNA assays from Applied Biosystems, in accordance with the manufacturer’s protocol. The expression levels were normalized to a reference gene (RNU6B) by using the 2^−ΔΔCT^ method. Analyses were performed on six independent experiments, each in triplicate.

### 2.10 miRNA and total RNA isolation from liver, skeletal muscle and vWAT and RT-qPCR

Liver, skeletal muscle and vWAT samples were homogenized using a polytron in an appropriate volume of QIAzol lysis buffer (QIAGEN, Hilden, Germany). miRNA and RNA were extracted by the miRNeasy mini kit (QIAGEN). Total RNA (1 µg) was used to synthesize cDNA strands in a 20-µL-reaction volume using the SuperScript IV reverse Transcriptase for RT-PCR (Invitrogen). 50µM of random hexamers, 10 mM of dNTP mix and 1 µg of total RNA were combined and heated at 65°C for 5 min and then incubated on ice for at least 1 min. Annealed RNA was combined with RT reaction mix and incubated at 23°C for 10 min, 50°C–55°C for 10 min and 80°C for 10 min. Real-Time quantitative RT-PCR (QRT-PCR) was conducted with 50 nM gene-specific primers, IQ SYBR Green supermix (Bio-Rad), and cDNA samples (2 µL) in a total volume of 25 µL. A melting curve analysis was completed following amplification from 55°C to 95°C to ensure product identification and homogeneity. The mRNA expression levels were repeated in triplicate and were normalized to a reference gene (B-ACTIN, GAPDH and Alpha-TUBULIN, stable under specific experimental conditions) by using the 2^−ΔΔCT^ method. PCR primers were designed by using the Primer 3 program (Untergasser et al., 2012), and synthesized and verified by sequencing at Eurofins Genomics (Ebersberg, Germany). miR-122-5p, miR-34a-5p, and miR-125b-5p were quantified along with RNU6B (reference transcript) by RT-qPCR with TaqMan^®^ miRNA assays from Applied Biosystems, in conformity with the manufacturer’s protocol. The expression levels were normalized to a reference gene (RNU6B) by using the 2^−ΔΔCT^ method. The analyses were performed on six independent experiments, each in triplicate.

Primers used were as follows:

PTPN1 forward: 5′- CTG​ACA​CCT​GCC​TCT​TAC​TG-3′

PTPN1 reverse: 5′-CAC​TTG​ACT​GGG​CTC​TGC-3′

SCD1 forward: 5′-CAG​TTC​CTA​CAC​GAC​CAC​CAC​TA-3′

SCD1 reverse: 5′-GGA​CGG​ATG​TCT​TCT​TCC​AGA​T-3′

DGAT-1 forward: 5′-GGA​CAA​AGA​CCG​GCA​GAC​CA-3′

DGAT-1 reverse: 5′-CAG​CAT​CAC​CAC​GCA​CCA​AT-3′

AGPAT-1 forward: 5′-CCT​CGA​CCT​GCT​TGG​AAT​GAT​GG-3′

AGPAT-1reverse: 5′-CAC​CTC​GGA​CAT​GAC​ACT​GAT​AGC-3′

SIRT-1 forward: 5′-AGC​ATC​ACA​CGC​AAG​CTC​TA-3′

SIRT-1 reverse: 5′-GTG​CCA​ATC​ATG​AGG​TGT​TG-3′

SREBP1c forward: 5′–GGC​CCT​GTG​TGT​ACT​GGT​CT-3′

SREBP1c reverse: 5′-AGC​ATC​AGA​GGG​AGT​GAG​GA-3′

SREBP2 forward: 5′-GCA​ACA​ACA​GCA​GTG​GCA​GAG-3′

SREBP2 reverse: 5′-TGA​GGG​AGA​GAA​GGT​AGA​CAA​TGG-3′

ChREBP forward: 5′-CAG​GAT​GCA​GTC​CCT​GAA​AT-3′

ChREBP reverse: 5′-GAG​GTG​GCC​TAG​GTG​GTG​TA-3′

FAS forward: 5′-CAC​GGC​GGC​AGC​AGG​AAC​AG -3′

FAS reverse: 5′-AGC​ACT​CTC​AGA​CAG​GCA​CTC​AG-3′

HMGCoA red forward: 5′-CGACAGCA GAGCAGATTTG-3′

HMGCoA red reverse: 5′-TGG​ACT​GGA​GAC​GGA​TGT​AGA​G-3′

ACC forward: 5′-GAC​GTT​CGC​CAT​AAC​CAA​GT-3′

ACC reverse: 5′-CTG​CAG​GTT​CTC​AAT​GCA​AA-3′

SPOT14 forward: 5′-CTG​AGG​AAG​ACA​GGC​TTT​CG-3′

SPOT14 reverse: 5′-TTC​TGG​GTC​AGG​TGG​GTA​AG-3′

INSIG1 forward: 5′-GGT​TCT​CGG​GTC​ATT​TCA​GA-3′

INSIG1 reverse: 5′-AGG​GTG​TAG​TGG​AGG​GTG​TG-3′

B-ACTIN forward: 5′-GGA​GAT​TAC​TGC​CCT​GGC​TCC​TA-3′

B-ACTIN reverse: 5′-GAC​TCA​TCG​TAC​TCC​TGC​TTG​CTG-3′

GAPDH forward: 5′-GCA​CCG​TCA​AGG​CTG​AGA​AC-3′

GAPDH reverse: 5′-TGG​TGA​AGA​CGC​CAG​TGG​A-3′

Alpha-TUBULIN forward: 5′-CCA​CTC​ATT​CCC​TCC​TTG​AA-3'

Alpha-TUBULIN reverse: 5′-ATG​GCT​CCA​TCA​AAC​CTC​AG-3'

### 2.11 Statistical analysis

All results were analyzed with the GraphPad Prism 9 software system (GraphPad Software, San Diego, CA, United States). Data were expressed as the mean ± SEM and were normally distributed. The statistical significance of the differences between experimental groups was determined using two-way ANOVA followed by the Tukey post-hoc test. Differences were considered statistically significant at *p* < 0.05.

## 3 Results

### 3.1 Short-term fructose feeding impairs insulin signaling and induces an inflammatory state in young and adult rats without affecting body weight gain

**FIGURE 1:**
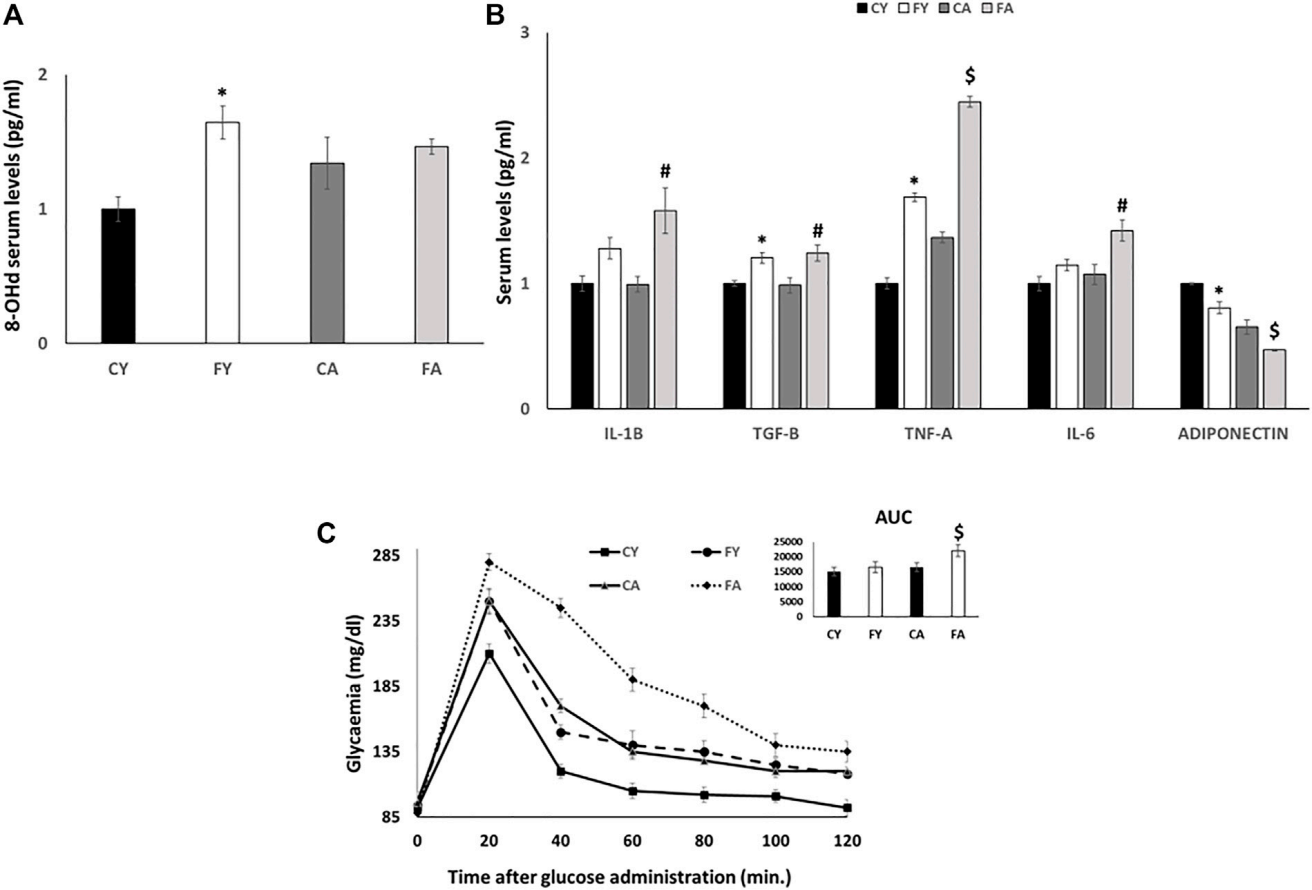
**(A)** 8-OHdG serum levels (pg/ml) detected by a competitive Enzyme Linked-Immunosorbent Assay (ELISA). **(B)** IL-1B, TGF-B, TNF-A, IL-6 and ADIPONECTIN serum levels (pg/ml) detected by a competitive Enzyme Linked-Immunosorbent Assay (ELISA). **(C)** Whole body glucose homeostasis in young and adult rats fed a control or fructose-rich diet for 2 weeks. The day before euthanasia, rats were fasted for 6 h, fasting blood samples were collected and then rats received an intraperitoneal injection of glucose (2 g/kg b.w.). Aliquots of blood were taken at 20, 40, 60, 80, 100, and 120 min after injection and used for the determination of plasma glucose concentration. The area under the curve (AUC) of plasma glucose during glucose load was calculated with the trapezoid method. Values are the means ± SEM of six different rats (*n* = 6). Values with different symbols are significantly different: *p < 0.05 vs. CY, #*p* < 0.05 vs. CA; $*p* < 0.05 vs. FY and CA (*p* < 0.05, Tukey post-test). CY: young rats receiving a standard diet for 2 weeks; FY: young rats receiving a fructose-rich diet for 2 weeks; CA: adult rats receiving a standard diet for 2 weeks; FA: adult rats receiving a fructose-rich diet for 2 weeks. Two-way ANOVA p results: **(A)** Diet effect = 0.0345, age effect = 0.0569. **(B)** Diet effect = 0.0041 and age effect = 0.2175 for IL-1B, diet effect = 0.0018 and age effect = 0.8068 for TGF-B, diet effect = 0.0019 and age effect= <0.0001 for TNF-A, diet effect = 0.0451 and age effect = 0.0845 for IL-6; diet effect = 0.0014 and age effect = 0.0001 for ADIPONECTIN. **(C)** Diet effect = 0.0012, age effect = 0.00013.

Following 2 weeks of dietary treatment, body weight gain and tissue weight were measured. The results revealed no increase in body weight gain in rats fed on HFrD compared to the control diet. No differences were observed regarding weight of liver, skeletal muscle, and vWAT in FY or FA rats with the exception of FA vWAT, which increased significantly compared with CA. Serum triglycerides were significantly elevated in FY and FA compared to the control group (Table 2). An increase was observed in the serum levels of 8-hydroxy-2′-deoxyguanosine (8-OHdG), an oxidized derivative of the guanine base considered as a marker of systemic oxidative stress (Cheng et al., 1992; Grollman and Moriya, 1993; Di Minno et al., 2016), in FY and FA compared to control rats (Figure 1A). Young and adult rats fed on HFrD showed an increase in serum levels of Transforming growth factor beta (TGF-B), Interleukin-1 beta (IL-1B), Tumor necrosis factor alpha (TNF-A) and Interleukin-6 (IL-6), with lower serum adiponectin levels in relation to the rats fed on control diet (Figure 1B). These data highlight an increase in systemic oxidative stress, the establishment of an inflammatory state and the impairment of insulin signaling induced by fructose. The impairment of glycemic homeostasis was confirmed by performing the oral glucose tolerance test (OGTT) (Figure 1C). Adult rats fed on HFrD (FA) showed a reduced glucose tolerance compared to CA and FY animals and showed a significantly higher area under the curve (AUC) value of plasma glucose in comparison to both CA and FY groups (Figure 1C).

**TABLE 2:** Body weight gain, liver weight, skeletal muscle weight, vWAT weight and serum triglycerides levels in CY, FY, CA, and FA rats.

	Young		Adult		Two-way ANOVA *p*-values	
	Control	Fructose	Control	Fructose	Diet effect	Age effect
Body weight gain, g	113 ± 2	118 ± 2	45 ± 10	53 ± 10	0.2374	0.4261
Liver, g	12.1 ± 0.7	11.7 ± 0.5	17.0 ± 1.0	18.1 ± 0.9	0.3112	0.4376
Skeletal muscle, g	6.3 ± 0.2	6.1 ± 0.1	8.9 ± 0.3	9.0 ± 0.4	0.1487	0.6864
vWAT, g	1.1 ± 0.1	1.1 ± 0.1	4.9 ± 0.2	5.9 ± 0.2^#^	0.0467	0.0495
Serum triglycerides (mg/100 mL)	74.7 ± 4.8	128.3 ± 7.3*	145.3 ± 19.8	222 ± 9.1^$^	<0.0001	0.0045

Body weight gain (g), liver weight (g), skeletal muscle weight (g), vWAT weight (g), and serum triglycerides (mg/100 ml) in CY, FY, CA, and FA rats. CY: Young rats receiving a standard diet for 2 weeks; FY: Young rats receiving a fructose-rich diet for 2 weeks; CA: Adult rats receiving a standard diet for 2 weeks; FA: Adult rats receiving a fructose-rich diet for 2 weeks. Values are the means ± SEM of six different rats (*n* = 6). Values with different symbols are significantly different: *p < 0.05 vs. CY; #*p* < 0.05 vs. CA; $*p* < 0.05 vs. FY and CA (*p* < 0.05, Tukey post-test). CY: Young rats receiving a standard diet for 2 weeks; FY: Young rats receiving a fructose-rich diet for 2 weeks; CA: Adult rats receiving a standard diet for 2 weeks; FA: Adult rats receiving a fructose-rich diet for 2 weeks.

### 3.2 Short term fructose intake modulates miR-122-5p and miR-34a-5p expression

**FIGURE 2:**
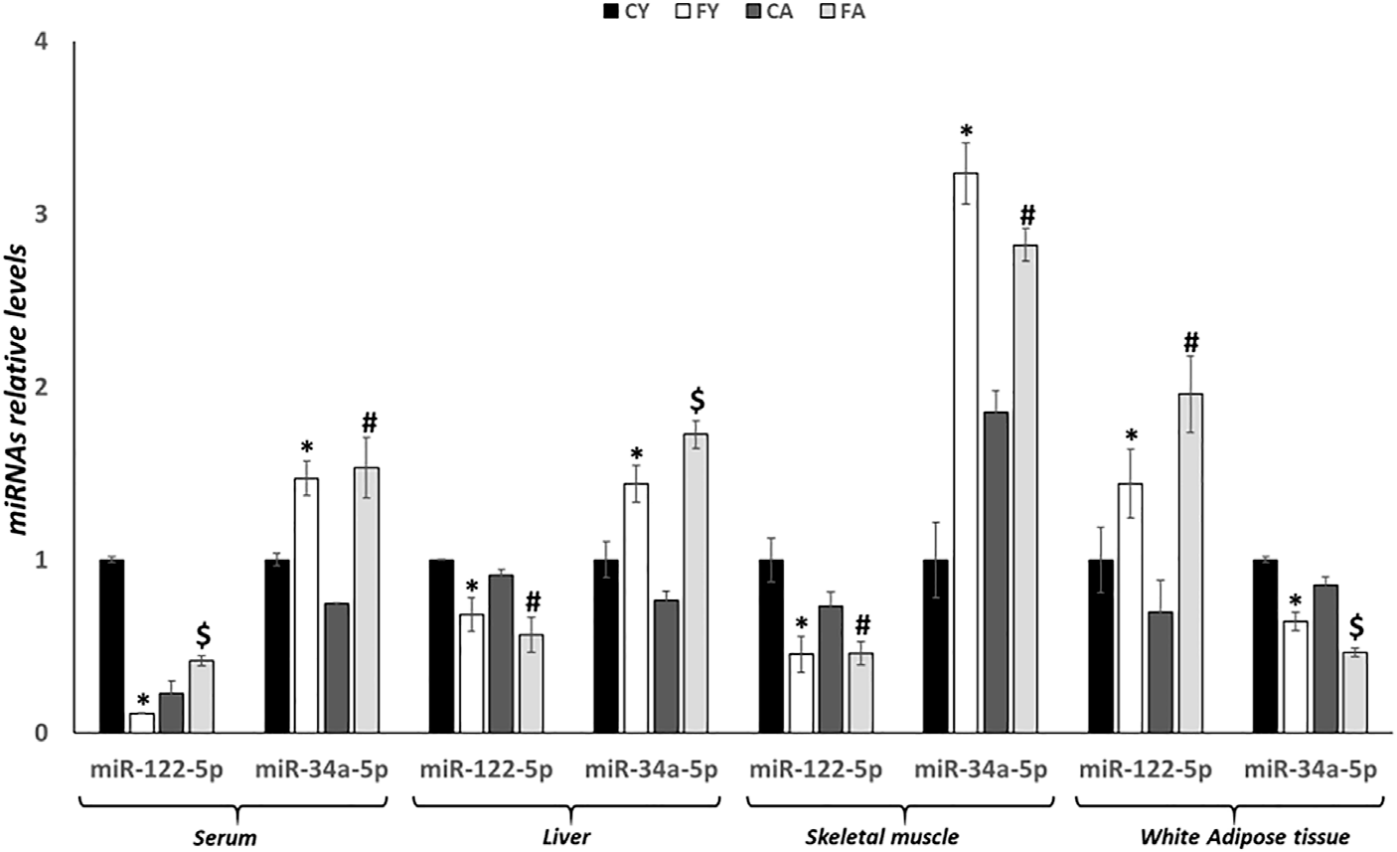
RT-qPCR analysis of miR-122-5p and miR-34a-5p in serum, liver, skeletal muscle and vWAT samples of young and adult rats fed on with a control or fructose-rich diet for 2 weeks. Values are the means ± SEM of six different rats (*n* = 6). Values with different symbols are significantly different: *p < 0.05 vs. CY; #*p* < 0.05 vs. CA; $*p* < 0.05 vs. FY and CA (*p* < 0.05, Tukey post-test). CY: young rats receiving a standard diet for 2 weeks; FY: young rats receiving a fructose-rich diet for 2 weeks; CA: adult rats receiving a standard diet for 2 weeks; FA: adult rats receiving a fructose-rich diet for 2 weeks. Two-way ANOVA p results: diet effect = 0.0013 and age effect = 0.0695 for serum miR-122-5p, diet effect = 0.0007 and age effect = 0.5406 for serum miR-34a-5p, diet effect = 0.0008 and age effect = 0.0768 for hepatic miR-122-5p, diet effect= <0.0001 and age effect = 0.5336 for hepatic miR-34a-5p, diet effect = 0.0004 and age effect = 0.0156 for skeletal muscle miR-122-5p, diet effect = 0.0006 and age effect = 0.2473 for skeletal muscle miR-34a-5p, diet effect = 0.0113 and age effect = 0.6392 for white adipose tissue miR-122-5p, diet effect = 0.0013 and age effect= <0.0001 for white adipose tissue miR-34a-5p.

As shown in Figure 2, miR-122-5p expression levels are significantly reduced in the liver and skeletal muscle of FY and FA rats, conversely, miRNA expression was significantly upregulated in adipose tissue concerning FY and FA groups when compared with CY and CA rats. In addition, miR-122-5p expression levels were significantly reduced in serum of the FY group when compared to CY animals but were markedly increased in FA rat serum in relation to the CA group.

Alternatively, miR-34a-5p expression levels were significantly increased in liver, skeletal muscle and serum of FY and FA rats, but were downregulated in adipose tissue of these animals (Figure 2). Furthermore, remarkable alterations in the liver and in the vWAT were also observed between young and adult animals fed on HFrD, suggesting age-related effects induced by fructose overconsumption (Figure 2).

### 3.3 Fructose administration impairs insulin signaling and lipid metabolism in liver and skeletal muscle by targeting miR-122-5p and miR-34a-5p

**FIGURE 3:**
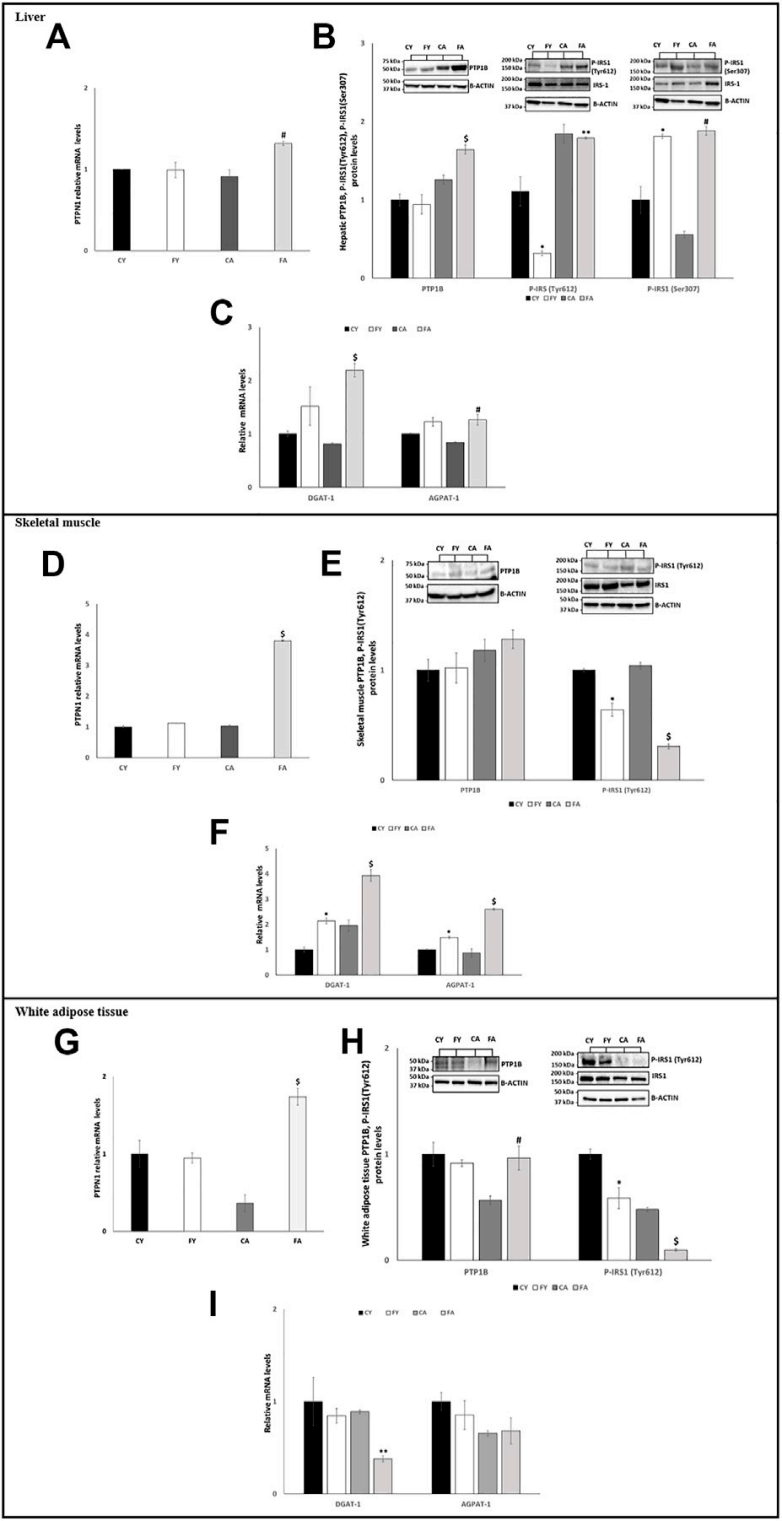
**(A,D,G)** RT-qPCR analysis of PTPN1 in liver, skeletal muscle and vWAT samples of young and adult rats fed on with a control or fructose-rich diet for 2 weeks. **(B)** Western blot images and densitometry of PTP1B, P-IRS1(Tyr612), and P-IRS1(Ser307) in liver samples of young and adult rats fed on with a control or fructose-rich diet for 2 weeks. **(C,F,I)** RT-qPCR analysis of DGAT-1 and AGPAT-1 in liver, skeletal muscle and vWAT samples of young and adult rats fed on with a control or fructose-rich diet for 2 weeks. **(E,H)** Western blot images and densitometry of PTP1B and P-IRS1(Tyr612) in skeletal muscle and vWAT samples of young and adult rats fed on with a control or fructose-rich diet for 2 weeks. Values are the means ± SEM of six different rats (*n* = 6). Representative blots are shown. Values with different symbols are significantly different *p < 0.05 vs. CY; #*p* < 0.05 vs. CA; $*p* < 0.05 vs. FY and CA; **p < 0.05 vs. FY (*p* < 0.05, Tukey post-test). CY: young rats receiving a standard diet for 2 weeks; FY: young rats receiving a fructose-rich diet for 2 weeks; CA: adult rats receiving a standard diet for 2 weeks; FA: adult rats receiving a fructose-rich diet for 2 weeks. Two-way ANOVA p results: **(A)** Diet effect = 0.1963, age effect = 0.0504. **(B)** Diet effect = 0.2387 and age effect = 0.0108 for PTP1B, diet effect = 0.003 and age effect = 0.0069 for P-IRS1(Tyr612), diet effect = 0.0056 and age effect = 0.009 for P-IRS1(Ser307). **(C)** Diet effect = 0.0012 and age effect = 0.0392 for DGAT-1, diet effect = 0.0058 and age effect = 0.5955 for AGPAT-1. **(D)** Diet effect= <0.0001, age effect= <0.0001. **(E)** Diet effect = 0.7581 and age effect = 0.8143 for PTP1B, diet effect = 0.0001 and age effect = 0.0151 for P-IRS1 (Tyr612). **(F)** Diet effect = 0.0009 and age effect = 0.0015 for DGAT-1, diet effect= <0.0001 and age effect = 0.0010 for AGPAT-1. **(G)** Diet effect = 0.0953, age effect = 0.0327. **(H)** Diet effect = 0.0833 and age effect = 0.0417 for PTP1B, diet effect = 0.0021 and age effect = 0.0008 for P-IRS1(Tyr612). **(I)** Diet effect = 0.0085 and age effect = 0.0096 for DGAT-1, diet effect = 0.1423 and age effect = 0.1239 for AGPAT-1.

First, we analyzed the miR-122-5p/PTP1B/P-IRS-1 (Tyr612) axis. Our results showed that, in rats fed on HFrD regardless of age, the expression levels of hepatic and skeletal muscle miR-122-5p were significantly decreased when compared with rats fed on standard diet (CY and CA) (Figure 2). PTP1B protein levels and PTPN1 mRNA expression levels were significantly upregulated in the liver of FA animals compared to the CA group while, in skeletal muscle, only PTPN1 mRNA expression levels were notably increased (Figures 3A, B, D). PTP1B catalyzes IRS1 de-phosphorylation, thus we measured the protein levels of P-IRS1(Tyr612). The results revealed that these protein levels were significantly reduced in liver and skeletal muscle of FY rats (Figures 3B, E). It is noteworthy to report differing results in liver and skeletal muscle of FA group; in skeletal muscle the P-IRS1(Tyr612) protein levels were remarkably decreased compared to both CA animals and FY animals (Figure 3E). In the liver of FA, however, the P-IRS1(Tyr612) a slight decrease was observed in the protein levels in relation to the CA animals (not significant) (Figure 3B). To better analyze insulin sensitivity in the liver, we measured the P-IRS1(Ser307) protein levels, being Ser307 phosphorylation related to insulin-resistance (Paz et al., 1996; Paz et al., 1999). The outcomes revealed that in liver of FY and FA animals the P-IRS1(Ser307) protein levels were markedly increased in relation to the control groups (Figure 3B).

Furthermore, miR-122-5p targets the 3′-UTR of Diacylglycerol O-Acyltransferase 1 (DGAT-1) and 1-Acyl-sn-Glycerol-3-Phosphate Acyltransferase Alpha (AGPAT-1), two genes involved in triglyceride synthesis (Chai et al., 2017). Our results showed that the mRNA expression levels of these two genes were significantly higher in liver of FA compared to CA and FY animals and in skeletal muscle of both FY and FA rats in comparison to the control groups and in relation to each other (Figures 3C, F).

**FIGURE 4:**
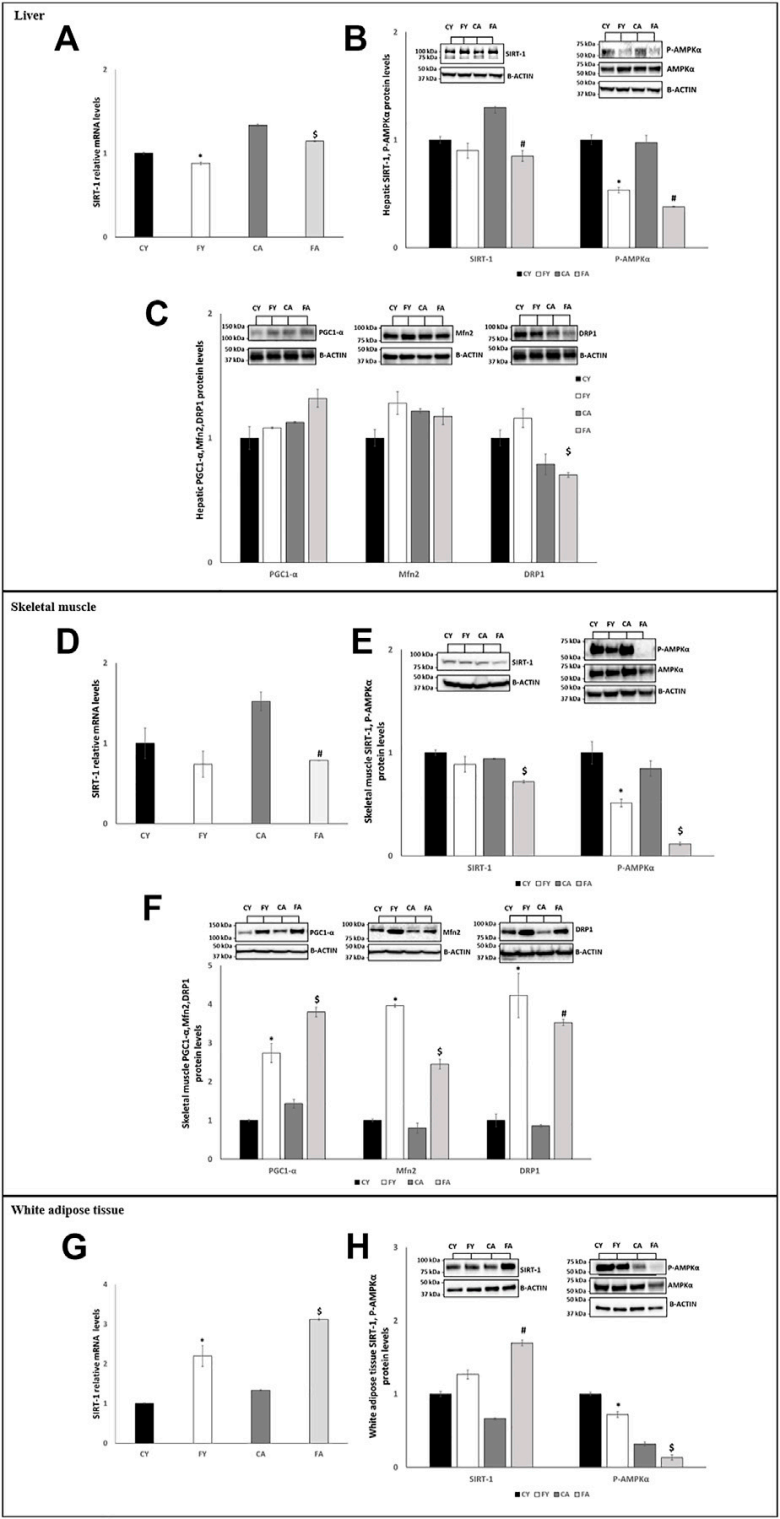
**(A,D,G)** RT-qPCR analysis of SIRT-1 in liver, skeletal muscle and vWAT samples of young and adult rats fed on with a control or fructose-rich diet for 2 weeks. **(B,E,H)** Western blot images and densitometry of SIRT-1 and P-AMPKα in liver, skeletal muscle and vWAT samples of young and adult rats fed on with a control or fructose-rich diet for 2 weeks; **(C,F)** Western blot images and densitometry of PGC1-α, Mfn2 and DRP1in liver and skeletal muscle samples of young and adult rats fed on with a control or fructose-rich diet for 2 weeks. Values are the means ± SEM of six different rats (*n* = 6). Representative blots are shown. Values with different symbols are significantly different *p < 0.05 vs. CY; #*p* < 0.05 vs. CA; $*p* < 0.05 vs. FY and CA (*p* < 0.05, Tukey post-test). CY: young rats receiving a standard diet for 2 weeks; FY: young rats receiving a fructose-rich diet for 2 weeks; CA: adult rats receiving a standard diet for 2 weeks; FA: adult rats receiving a fructose-rich diet for 2 weeks. Two-way ANOVA p results: **(A)** Diet effect = 0.007, age effect = 0.6905. **(B)** Diet effect = 0.0007 and age effect = 0.6905 for SIRT-1, diet effect = 0.0002 and age effect = 0.1032 for P-AMPKα. **(C)** Diet effect = 0.0808 and age effect = 0.0712 for PGC1-α, diet effect = 0.1345 and age effect = 0.5612 for Mfn2, diet effect = 0.0512 and age effect = 0.0459 for DRP1. **(D)** Diet effect = 0.0008, age effect = 0.0412. **(E)** Diet effect = 0.0006 and age effect = 0.0123 for SIRT-1, diet effect = 0.0008 and age effect = 0.0219 for P-AMPKα. **(F)** Diet effect = 0.0002 and age effect = 0.0758 for PGC1-α, diet effect= <0.0001 and age effect = 0.0008 for Mfn2, diet effect = 0.0006 and age effect = 0.2349 for DRP1. **(G)** Diet effect = 0.0453, age effect = 0.6193. **(H)** Diet effect = 0.0014 and age effect = 0.7093 for SIRT-1, diet effect = 0.0013 and age effect= <0.0001 for P-AMPKα.

In adipose tissue, the expression of miR-122-5p was significantly increased both in FY than in FA rats as compared to the control groups (Figure 2). Despite the increase in miR-122-5p expression, the PTP1B levels were unaltered in FY animals and significantly increased in FA rats compared to CA group (Figures 3G, H). In addition, the levels of P-IRS1 (Tyr612) were significantly reduced in FY and FA compared to the control groups (Figure 3H). The increase in miR-122-5p expression resulted in a decrease in DGAT-1 and AGPAT-1 expression levels in FY and FA groups (Figure 3I). Subsequently, we investigated the functional role of the miR-34a-5p/SIRT-1: AMPK pathway. Our results demonstrated that in animals fed on HFrD, the expression levels of miR-34a-5p were significantly increased in liver and skeletal muscle of FY and FA rats compared with CY and CA animals (Figure 2). Hepatic and skeletal muscle SIRT-1 expression levels and protein levels were unchanged in FY rats, in relation to CY, while in FA rats such levels were significantly downregulated in comparison to CA group (Figures 4A, B, D, E). P-AMPK levels were considerably reduced in both FY and FA in respect of the control groups (Figures 4B, E). Furthermore, in skeletal muscle, both SIRT-1 and P-AMPK protein levels were significantly reduced in FA rats when compared to FY animals (Figure 4E). Activation of the miR-34a-5p/SIRT-1:AMPK pathway has been shown to cause mitochondrial dynamic dysfunction (Simao et al., 2019), we thus measured Peroxisome Proliferator-Activated Receptor-gamma Coactivator (PGC1-α), implicated in mitochondrial biogenesis, Mitofusin-2 (Mnf2) and Dynamin-Related Protein 1 (DRP1) protein levels, involved in mitochondrial fusion and fission, both in liver and in skeletal muscle.

Our findings revealed that in liver of FY rats, the levels of PGC1-α, Mnf2, and DRP1 increased by approximately about 8%, 28%, and 16%, respectively (not significant) when compared with CY animals (Figure 4C). In liver of FA animals, the levels of PGC1-α increased by approximately 18% (not significant), Mnf2 levels reduced by approximately 4% (not significant) and DRP1 levels significantly decreased by approximately 11% in comparison to CA (Figure 4C).

Conversely, in skeletal muscle of both FY and FA, the PGC1-α, Mnf2, and DRP1 protein levels were more than two-fold higher in relation to the control groups (Figure 4F).

In vWAT, we observed a diverse regulation of miR-34a-5p expression. In fact, in adipose tissue, miR-34a-5p expression levels were significantly decreased in HFrD-fed animals (Figure 2) with its downstream target SIRT-1 significantly increased both in FY and FA (Figures 4G, H). The P-AMPK levels were reduced in FY and FA in comparison to the control groups and to each other (Figure 4H).

### 3.4 Effect of fructose administration on respiratory chain complexes, cytochrome oxidase (COX) activity and antioxidant enzymes in liver and skeletal muscle

**FIGURE 5:**
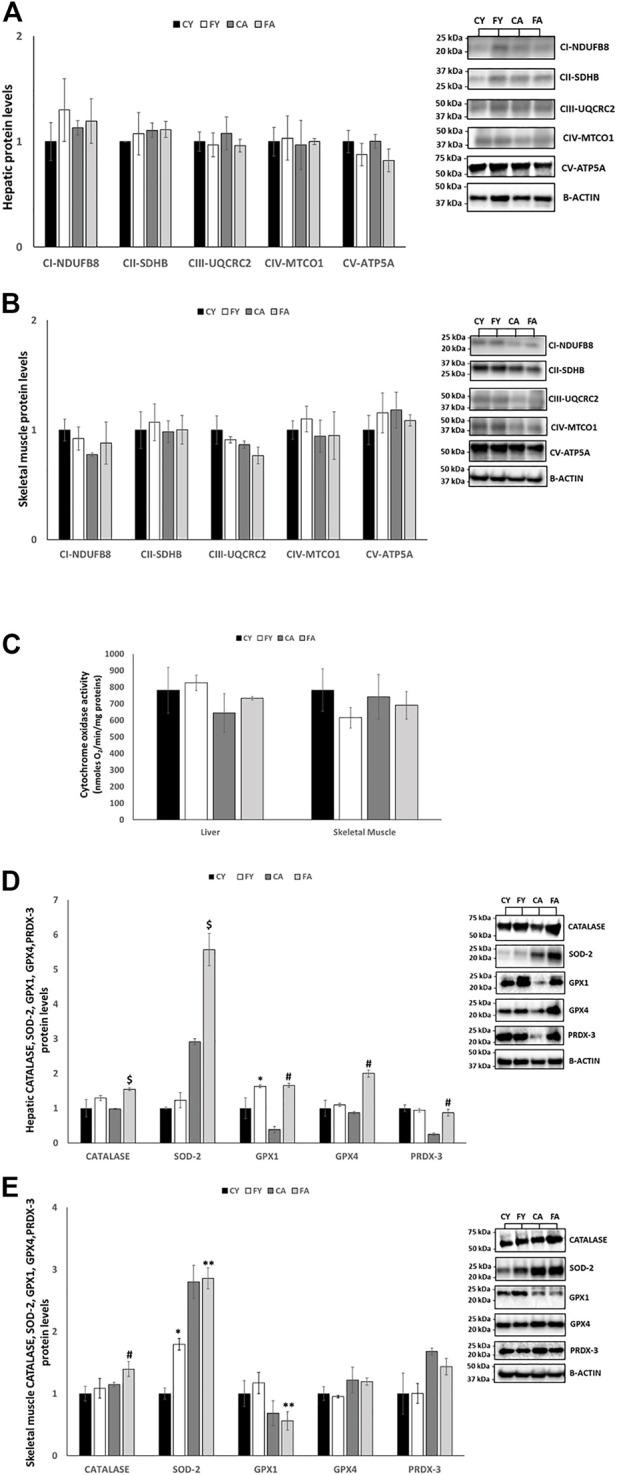
**(A,B)** Western blot images and densitometry of CI–CV respiratory chain complex in liver and skeletal muscle samples of young and adult rats fed on with a control or fructose-rich diet for 2 weeks. **(C)** Activity of cytochrome oxidase in liver and skeletal muscle homogenates of young and adult rats fed on with a control or fructose-rich diet for 2 weeks. **(D,E)** Western blot images and densitometry of CATALASE, SOD-2, GPX1, GPX4, and PRDX-3 in liver and skeletal muscle samples of young and adult rats fed on with a control or fructose-rich diet for 2 weeks. Values are the means ± SEM of six different rats (*n* = 6). Representative blots are shown. Values with different symbols are significantly different *p < 0.05 vs. CY; #*p* < 0.05 vs. CA; $*p* < 0.05 vs. FY and CA; **p < 0.05 vs. FY (*p* < 0.05, Tukey post-test). CY: young rats receiving a standard diet for 2 weeks; FY: young rats receiving a fructose-rich diet for 2 weeks; CA: adult rats receiving a standard diet for 2 weeks; FA: adult rats receiving a fructose-rich diet for 2 weeks. Two-way ANOVA p results: **(A)** Diet effect = 0.2567 and age effect = 0.4561 for CI-NDUFB8, diet effect = 0.3409 and age effect = 0.6548 for CII-SDHB, diet effect = 0.8769 and age effect = 0.5123 for CIII-UQCRC2, diet effect = 0.7845 and age effect = 0.1287 for CIV-MTCO1, diet effect = 0.6521 and age effect = 0.0945 for CV-ATP5A. **(B)** Diet effect = 0.0912 and age effect = 0.5148 for CI-NDUFB8, diet effect = 0.5986 and age effect = 0.3243 for CII-SDHB, diet effect = 0.5412 and age effect = 0.8921 for CIII-UQCRC2, diet effect = 0.9921 and age effect = 8,832 for CIV-MTCO1, diet effect = 0.4432 and age effect = 0.5513 for CV-ATP5A. **(C)** Liver: Diet effect = 0.3524, age effect = 0.2345; Skeletal muscle: diet effect = 0.3145, age effect = 0.2697. **(D)** Diet effect = 0.0100 and age effect = 0.4941 for CATALASE, diet effect = 0.0337 and age effect= <0.0001 for SOD-2, diet effect = 0.0042 and age effect = 0.1415 for GPX1, diet effect = 0.0007 and age effect = 0.0071 for GPX4, diet effect = 0.2150 and age effect = 0.0057 for PRDX-3. **(E)** Diet effect = 0.0406 and age effect = 0.2060 for CATALASE, diet effect = 0.0409 and age effect = 0.0003 for SOD-2, diet effect = 0.7215 and age effect = 0.0189 for GPX1, diet effect = 0.8024 and age effect = 0.1526 for GPX4, diet effect = 0.6392 and age effect = 0.0625 for PRDX-3.

To further evaluate mitochondrial modifications elicited by short-term fructose overconsumption, we examined respiratory chain complexes (CI-CV), cytochrome oxidase (COX) activity and antioxidant enzymes in the liver and skeletal muscle. In the liver of FY and FA rats, the protein levels of respiratory chain complexes CIII and CV were slightly reduced and the COX activity was increased in FY and FA group by approximately 6% and 14% (not significantly), respectively (Figure 5C). In skeletal muscle, the protein levels of subunits of respiratory chain complexes CI–CV were unaltered considering the experimental groups (Figures 5A, B) and the COX activity was reduced in FY and FA group by approximately 7% and 21% (not significantly), respectively.

As reported in Figure 5C, in liver, the protein levels of antioxidant enzymes such as catalase (CAT), superoxide dismutase 2 (SOD-2), glutathione peroxidase 1 (GPX1), glutathione peroxidase 4 (GPX4) and peroxiredoxin 3 (PRDX-3) were significantly increased in FY and FA rats compared to their control groups (Figure 5D). In skeletal muscle, the protein levels of SOD-2 were markedly increased in FY animals compared to CY group and in FA rats compared to FY animals. The protein levels of CAT were considerably increased in FA animals compared to their control group and GPX1 levels were decreased in FA group compared to FY animals. The protein levels of GPX4, and PRDX-3 remained unmodified albeit with a mild increase in animals treated with fructose (Figure 5E).

### 3.5 Fructose administration stimulates *de novo* lipogenesis in liver by targeting miR-125b-5p

**FIGURE 6:**
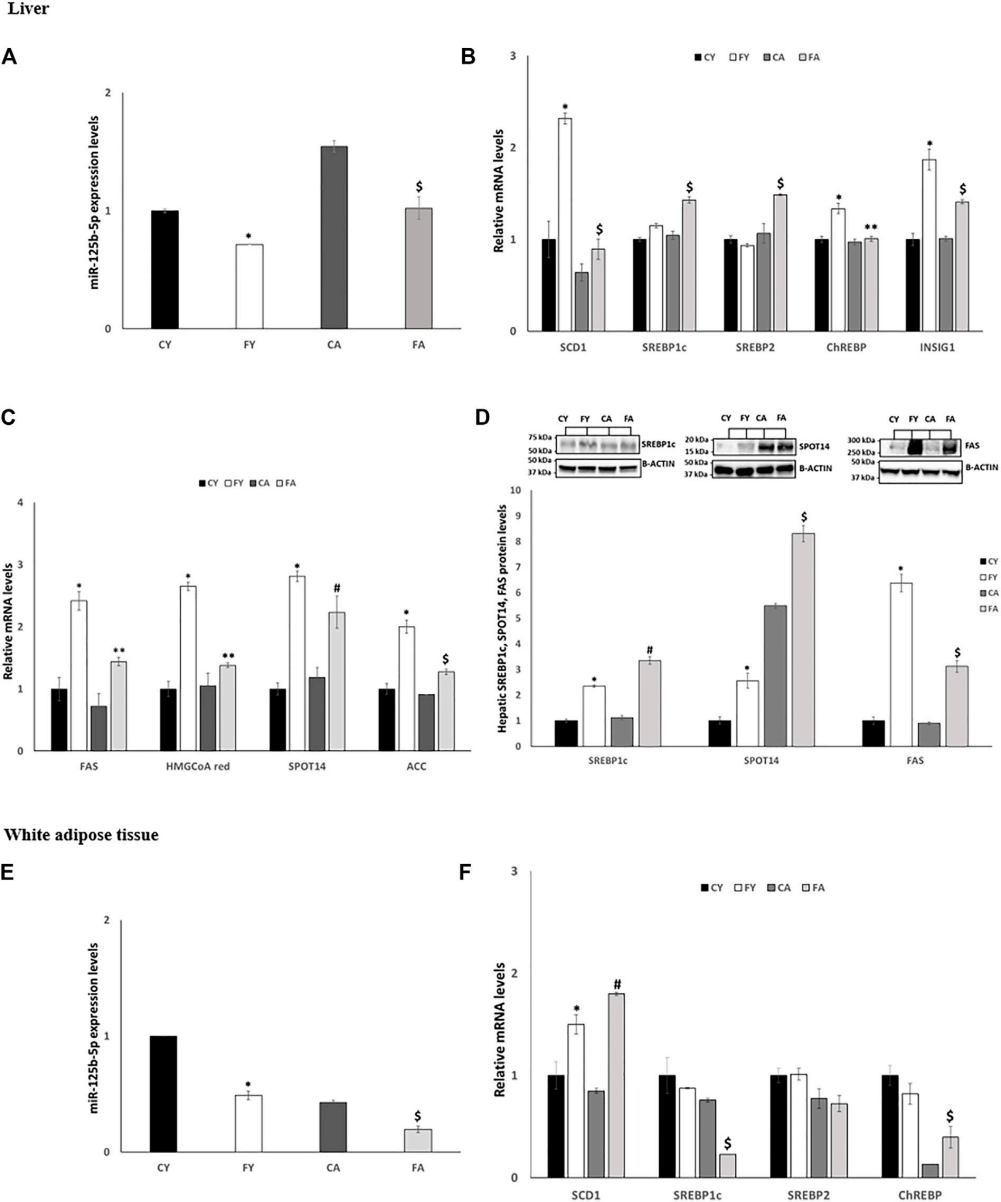
**(A,E)** RT-qPCR analysis of miR-125b-5p in liver and vWAT samples of young and adult rats fed on with a control or fructose-rich diet for 2 weeks. **(B)** RT-qPCR analysis of miR-125b-5p target genes in liver samples of young and adult rats fed on with a control or fructose-rich diet for 2 weeks. **(C)** RT-qPCR analysis of genes involved in fatty acid biosynthesis in liver samples of young and adult rats fed on with a control or fructose-rich diet for 2 weeks. **(D)** Western blot images and densitometry of SREBP1c, SPOT14 and FAS in liver samples of young and adult rats fed on with a control or fructose-rich diet for 2 weeks; **(F)** RT-qPCR analysis of SCD1, SREBP1c, SREBP2, and ChREBP in vWAT samples of young and adult rats fed on with a control or fructose-rich diet for 2 weeks. Values are the means ± SEM of six different rats (*n* = 6). Representative blots are shown. Values with different symbols are significantly different *p < 0.05 vs. CY; #*p* < 0.05 vs. CA; $*p* < 0.05 vs. FY and CA; **p < 0.05 vs. FY (*p* < 0.05, Tukey post-test). CY: young rats receiving a standard diet for 2 weeks; FY: young rats receiving a fructose-rich diet for 2 weeks; CA: adult rats receiving a standard diet for 2 weeks; FA: adult rats receiving a fructose-rich diet for 2 weeks. Two-way ANOVA p results: **(A)** diet effect = 0.0001, age effect = 0.0001. **(B)** Diet effect = 0.0019 and age effect = 0.0115 for SREBP1c, diet effect = 0.0392 and age effect = 0.0065 for SREBP2, -diet effect = 0.0085 and age effect = 0.0096 for ChREBP, -diet effect = 0.0007 and age effect = 0.0295 for INSIG1, diet effect = 0.0034 and age effect = 0.0021 for SCD1. **(C)** Diet effect = 0.0028 and age effect = 0.0179 for FAS, diet effect = 0.0013 and age effect = 0.0080 for HMGCoA red, diet effect = 0.0010 and age effect = 0.0039 for SPOT14, diet effect = 0.0007 and age effect = 0.0047 for ACC, **(D)** diet effect = 0.0023 and age effect = 0.0453 for SREBP1c, diet effect= <0.0001 and age effect= <0.0001 for SPOT14, diet effect= <0.0001 and age effect= <0.0001 for FAS. **(E)** Diet effect = 0.0002, age effect= <0.0001. **(F)** Diet effect = 0.0034 and age effect = 0.0021 for SCD1, diet effect = 0.0203 and age effect = 0.0072 for SREBP1c, diet effect = 0.8215 and age effect = 0.3303 for SREBP2, diet effect = 0.6845 and age effect = 0.0028 for ChREBP.

Short-term HFrD significantly decreases the expression levels of miR-125b-5p in liver and vWAT of young and adult when compared to control groups fed on standard diet. In addition, a significant increase in miR-125b-5p was observed in FA when compared to FY rats (Figures 6A, E). The expression levels of SCD1, SREBP1c, SREBP2 (Sterol Regulatory Element-Binding Protein 2) and INSIG1 (Insulin induced gene 1), all target of miR-125b-5p, were significantly increased in liver of FA compared to CA and FY animals (Figure 6B). The expression levels of ChREBP (Carbohydrate-Responsive Element-Binding Protein) were markedly upregulated in liver of FY compared to CY rats. The enhanced hepatic lipogenesis observed in HFrD-fed rats was also confirmed by the increased expression levels of numerous other genes involved in fatty acid biosynthesis as Fatty Acid Synthase (FAS), 3-Hydroxy-3-Methylglutaryl Coenzyme A (HMG-CoA), SPOT14 and Acetyl-CoA Carboxylase (ACC) (Figure 6C). In addition, the protein levels of SREBP1c, FAS and SPOT14, the major transcription factors that regulate genes involved in fatty acid synthesis (Porstmann et al., 2005) were significantly increased in both FY and FA rats compared to CY and CA animals (Figure 6D) and compared to each other. In vWAT, however, the expression levels of miR-125b-5p target genes showed a trend to decrease (Figure 6F) except for SCD1 whose expression was significantly increased in FY and FA animals compared with the control group (Figure 6F). Conversely, SREBP1c expression levels were reduced in FA compared to CA and FY groups and ChREBP expression levels were significantly decreased in FA group compared to FY rats (Figure 6F). Conversely, ChREBP expression levels were significantly increased in FA rats compared to CA animals (Figure 6F).

## 4 Discussion

A substantial worldwide increase in metabolic diseases in children and adolescents (DiStefano and Shaibi, 2021) requires in-depth studies on behalf of the scientific community to better understand the cellular mechanisms triggered by the excessive abuse of certain nutrients. In this perspective, herein, we investigated the involvement of miR-122-5p, miR-34a-5p, and miR-125b-5p and effects induced by fructose-overconsumption in young and adult rats, adding further knowledge as regards the molecular mechanisms underlying fructose-related metabolic alterations. We specifically focused on the early alterations and events occurring in young and adult rats, to highlight different age-dependent responses. Furthermore, among the many identified miRNAs involved in metabolism and metabolic diseases, miR-122-5p, miR-34a-5p, and miR-125b-5p possess specific expression patterns and functions associated with obesity, insulin resistance and inflammatory states (Li et al., 2011; Yang et al., 2012; Zhu and Leung, 2015; Cai et al., 2020). In fact, numerous studies have shown that miR-122-5p and miR-34a-5p expression levels are dysregulated and modulated in the development of NAFLD, Non-alcoholic steatohepatitis (NASH), and Hepatocellular carcinoma (HCC) (Tian et al., 2013; Gori et al., 2014; Tessitore et al., 2016; Hochreuter et al., 2022). As the result of miR-122-5p deletion, mice showed steatosis, fibrosis, and HCC, suggesting that this miRNA plays an essential role in NAFLD initiation and progression (Tsai et al., 2012). In addition, miR-122-5p could also play an active role in the diagnosis of NAFLD, since its plasma levels are higher in diabetic patients with NAFLD than in diabetics without this disease (Ye et al., 2018). Recently, a clinical study have examined the differential effects of circulating miRNAs, including miR-34a-5p and miR-122-5p, on histopathology and some clinical factors associated with NAFLD (Ezaz et al., 2020). Thus, herein, we aimed to clarify what occurs during the early stages of fructose administration in the three metabolic tissues.

**FIGURE 7:**
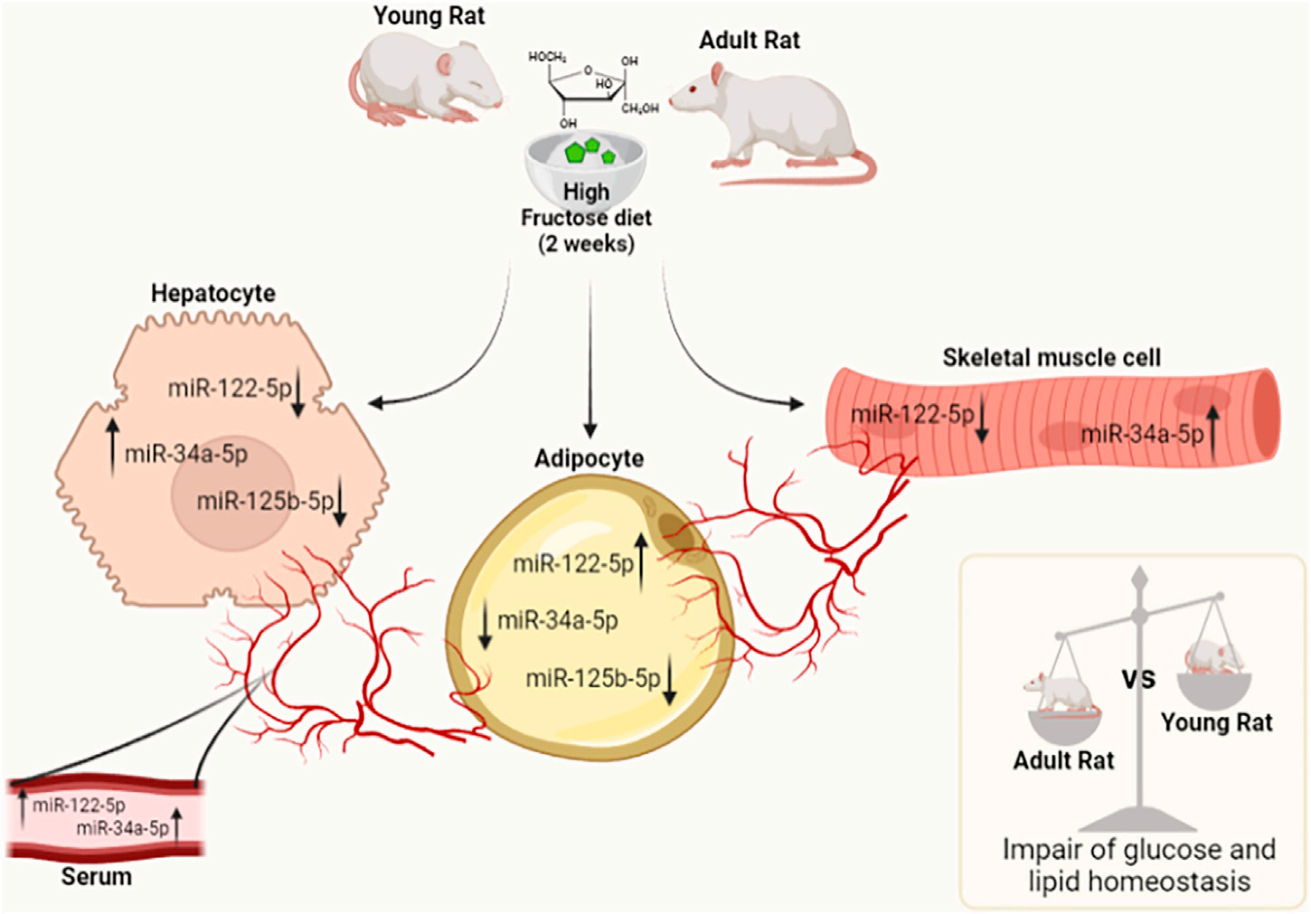
MiRNAs tissue trend in serum, liver, skeletal muscle, and vWAT in young and adult rats fed on HFrD (2 weeks). The modulation of studied miRNA levels shows a tissue-specific pattern, similar in the liver and skeletal muscle and conversely in vWAT. The results suggest miRNAs may serve as endocrine factors connecting different compartments and the circulatory system could allow for the specific type of crosstalk between tissues. The outcomes demonstrate that both young and adult rats fed on HFrD exhibit impaired glucose and lipid metabolism. In adult rats, this impairment is more pronounced than in young animals (The figure was created with Biorender.com).

Our findings suggest that both young and adult rats fed on HFrD exhibit an increase in systemic oxidative stress, the establishment of an inflammatory state, metabolic perturbations in liver, skeletal muscle and vWAT and that the assayed miRNAs and their target genes may play a critical role in impaired glucose and lipid homeostasis. Adult rats show a greater susceptibility to impaired insulin signaling when compared to young animals, while both young and adult rats fed on HFrD increase hepatic *de novo* lipogenesis (DNL), which leads to an excess of fat accumulation and mitochondrial dynamic dysfunction. In addition, liver and skeletal muscle of young and adult rats exhibit an imbalance in antioxidant enzyme. The modulation of studied miRNA levels shows a tissue-specific trend, similar in liver and skeletal muscle and dissimilar in vWAT. Such trends imply that miRNAs could act as endocrine factors connecting different compartments, with the circulatory system enabling a crosstalk between the various tissues (a schematic representation is displayed in Figure 7).

Short-term high fructose intake is sufficient to create systemic oxidative stress status and systemic low-grade metabolic inflammation that precedes the development of obesity. No increase was observed in body weight following high-fructose intake for 2 weeks. Nevertheless, the serum levels of 8-OHdG were higher in FY and FA with a more prominent increase in FY. Enhanced whole body oxidative stress likewise contributes to occurrence and aggravation of inflammation (Zhang et al., 2019; Ma et al., 2022). In fact, the present study reported a noteworthy rise in the expression levels of TGF-B and other key markers of systemic inflammation, namely IL-1B, TNF-A, and IL-6 in fructose-fed rats. In addition, an age-dependent increase in systemic inflammation was evident in both control and fructose-fed rats, in line with the biological phenomenon of “inflamm-aging” (Fougere et al., 2016).

The fructose overconsumption induces a condition of a systemic insulin-resistance both in young and adult rats. The aforementioned metabolic dysfunctions are more pronounced in adult rats suggesting that they are more susceptible to impairment in glucose homeostasis. Subsequently, to further acquire knowledge of the potential mechanisms involved in the effects of fructose on insulin sensitivity and lipid metabolism, we evaluated the involvement of miR-122-5p, miR-34a-5p, and miR-125b-5p and their downstream effectors in liver, skeletal muscle and vWAT.

miR-122-5p is the most abundant miRNA in hepatocytes accounting for approximately 70% of total adult liver miRNAs (Jopling, 2012) and it has been associated to metabolic hepatic homeostasis (Ceccarelli et al., 2013; Su et al., 2018; Dong et al., 2019b; Kalaki-Jouybari et al., 2020). Numerous metabolic processes are regulated by miR-122-5p, including cholesterol biosynthesis, fatty acid synthesis, and oxidation (Li et al., 2011). Interestingly, miR-122-5p is released from the liver under normal conditions, mainly *via* hepatic exosomes, a mechanism modulated by statins (Gallo et al., 2012; Huang et al., 2013). One of its direct downstream targets is PTP1B, a negative regulator of insulin signaling (Goldstein, 1993; Haeusler and Accili, 2008). In mice fed a high fat diet (HFD), miR-122-5p repression results in hepatic insulin resistance through the activation of PTP1B (Yang et al., 2012). In addition, a reduced expression of miR-122-5p was observed in weaned offspring (day 28) of mice fed a HFD compared to a standard chow diet (Benatti et al., 2014). Our results show that the miR-122-5p expression levels were significantly downregulated in the liver and in the skeletal muscle and upregulated in the vWAT of animals fed on HFrD. In the serum, miR-122-5p expression levels showed a different trend in young and adult rats fed on HFrD. The incongruent events occurring in FA regarding increased serum levels in miR-122-5p and decreased levels in liver are compliant with the outcomes of Baranova et al. (2019). A reduced liver secretion of miR-122-5p has been reported as the result of a decreased intra-hepatic production while miRNA production and secretion by adipose gland are able to enhance and maintain liver function. Our data suggest that the increased production of miR-122-5p by vWAT, may constitute effective counteraction to prevent early detrimental effects induced by fructose overconsumption. In this perspective, miR-122-5p could act as an “endocrine mediator” connecting different tissues.

miR-122-5p/PTP1B/P-IRS(Tyr612) axis plays a role in the alteration of insulin signaling induced by fructose overconsumption in skeletal muscle and vWAT of FA rats. In the liver of FA the increased P-IRS levels on Serine 307 confirms the impaired insulin response and insulin resistance occurring in the liver. In the FY group, the insulin signaling pathway impairment is apparently unrelated to the axis examined. In addition, the downregulation of miR-122-5p in liver and skeletal muscle of rats fed on HFrD yields a significant increase of the DGAT-1 and AGPAT-1 expression levels causing increased serum levels of triglycerides.

Fructose consumption, also affects miR-34a-5p/SIRT-1: AMPK pathway in liver and skeletal muscle of FY and FA rats inducing a decrease in fatty acid oxidation thus resulting in an increase in fat accumulation. miR-34a-5p is remarkably elevated in the liver of diet-induced animal models of NAFLD (Choi et al., 2013; Ding et al., 2015). The level of miR-34a-5p is increased in the liver of streptozotocin-induced diabetic mice and diet-induced obese mice compared with those of normal C57BL/6 mice (Li et al., 2009). Furthermore, miR-34a−/− mice are susceptible to diet-induced obesity (Lavery et al., 2016). In 2021, Xu et al. (2021) have demonstrated that, in hepatocytes, miR-34a-5p expression aggravates NAFLD induced by high-fat/cholesterol/fructose diets and that its deletion attenuates the development and progression of NAFLD. SIRT-1 is a direct target of miR-34a-5p (Castro et al., 2013; Dong et al., 2019a; Hua et al., 2021) and plays a beneficial role in regulating hepatic lipid metabolism through the deacetylation of certain transcriptional factors (Ding et al., 2017). Moreover, mice overexpressing SIRT-1 show elevated levels of AMPK, an indicator of metabolic status (Viollet et al., 2006; Ruderman et al., 2010). Recently, it has also been reported that in skeletal muscle the activation of the miR-34a/SIRT-1: AMPK pathway leads to mitochondrial dynamic dysfunction (Simao et al., 2019). Our results indicate that miR-34a-5p expression levels underline a tissue-specific regulation. In fact, in liver, skeletal muscle and serum, the miRNA expression levels are significantly increased in animals fed on HFrD while in vWAT the miR-34a-5p levels are significantly decreased in FY and FA when compared with their control group. Recently, skeletal muscle and adipose tissue have been shown to secrete miR-34a-5p that could act as an endocrine or paracrine mediator of inflammation and aging (Fulzele et al., 2019; Pan et al., 2019; Alfonzo et al., 2022). In our experimental conditions, the marked increase of miR-34a-5p, especially in skeletal muscle of both FY and FA groups, associated to its enhancement in serum levels suggest that miR-34a-5p could act as a mediator of inflammation.

In liver and skeletal muscle of FY and FA rats, the increase in miR-34a-5p levels leads to a reduction in SIRT-1 levels and in P-AMPK levels indicating a decrease of fat oxidation and an increase in fat synthesis. It is known that SIRT-1 affects mitochondrial function and dynamics, and as mitochondrial dysfunction characterizes many metabolic diseases, we evaluated the protein levels of PGC1-α, Mnf2 and DRP1, involved in biogenesis, fission and fusion, respectively. The results showed that 2 weeks of fructose overconsumption in young and adult animals were insufficient to alter mitochondrial dynamics in skeletal muscle, where an increase in biogenesis, fission and fusion was noted. In liver of adult rats, an increase in PGC1-α suggesting an enhanced biogenesis was evident. Conversely, the protein levels of Mnf2 and DRP1 in FA rats decreases indicating a reduction in fission and fusion. Considering that mitochondrial fission is an effector of ROS production in metabolic excesses (Galloway et al., 2014), the reduction in Mnf2 levels could counteract the progression of hepatic steatosis. To verify and confirm alteration in mitochondrial homeostasis, in the liver and skeletal muscle, we analyzed the oxidative stress. The mild decreased expression of certain respiratory chain complexes (CIII and CV) in FY and FA rats could impair ATP synthesis in the liver. This impairment may give rise to several vicious cycles involving ROS. Moreover, the increased lipogenesis and the decreased fatty acid *β*-Oxidation, lead to fat accumulation and insulin signaling impairment in the liver. In addition, the protein levels of key enzymes in protecting cells from oxidative damage, are increased in FY and FA animals promoting antioxidant defense, albeit insufficient to counter systemic oxidative stress. In the skeletal muscle, we observed a similar condition with an imbalance of antioxidant machinery.

In vWAT, miR-34a-5p levels were significantly decreased in FY and FA animals and the SIRT-1 expression levels and protein levels were increased. During normal eating conditions, SIRT-1 in adipose tissue protects against inflammation and obesity, and prevents metabolic dysfunction during dietary stress (Chalkiadaki and Guarente, 2012). In our study, we observed that in vWAT the increase in the SIRT-1 expression levels likely act to counteract the condition of insulin-resistance and inflammation that is established by the fructose overconsumption. Despite the increase in SIRT-1 levels, the P-AMPK levels were significantly decreased in vWAT of both FY and FA rats, highlighting a reduction in fat oxidation.

Finally, the miR-125b-5p was also seen to be modulated by HFrD. This miRNA is known to play an important role in DNL (Xie et al., 2009; Cheng et al., 2016) and miR-125b-5p knockout mice exhibit fat accumulation and insulin resistance induced by a high-fat diet (Sud et al., 2017; Wei et al., 2020). miR-125b-5p was downregulated in the liver of adult mice fed on HFrD for 4 weeks (Sud et al., 2017). The targets of this miRNA are the different key genes involved in insulin signaling, insulin resistance, fatty acid, triglyceride, lipoprotein and cholesterol biosynthesis and NAFLD. Cai et al. (2020) demonstrated that miR-125b-5p expression was reduced in NAFLD clinical samples, high cholesterol diet fed mice and the cell-model of NAFLD. We analysed the miR-125b-5p axis in liver and vWAT for their cooperative role played in lipid metabolism control. Our results show that short-term HFrD sufficed in significantly decreasing the miR-125b-5p expression levels in the liver and the vWAT of FY and FA rats compared to CY and CA animals and compared to each other. In the liver all the target genes of miR-125b-5p, were considerably expressed in FY and FA rats suggesting an increase in hepatic lipogenesis. The enhanced hepatic lipogenesis was also confirmed by the increased levels of several proteins involved in fatty acid biosynthesis.

In the vWAT, despite the reduction in miR-125b-5p in FY and FA rats, the expression levels of the miRNA target genes showed a trend to decrease, suggesting a reduction in DNL. This may be associated with pathophysiological events, such as obesity and insulin resistance, whereby the balance of DNL between hepatocytes and adipocyte is impeded (Song et al., 2018) diversely to physiological conditions in which DNL in hepatocytes and adipoctyes is synergistically regulated.

In conclusion, our findings highlight a strict involvement of some miRNAs in the metabolic dysregulations induced by short-term fructose overconsumption. A better understanding of dietary factors and modulation of miRNA expression levels and their targets could contribute to determine the molecular mechanisms underlying the metabolic diseases to identify novel therapeutic targets. These findings imply the need to reduce sugar consumption particularly during adolescence. In this perspective, our study lacked animal models consuming fructose on a long-term basis, thus presenting a limitation in the inability to assess miRNA modification over time. Additionally, in the light of developments herein, we hypothesize the potential of miR-122-5 p and miR34a-5p to mediate inter-tissue communication, hence, future studies would address the time course of exosomial secretion and content in young animals in response to long-term fructose consumption. Therefore, it is challenging to evaluate biological processes occurring in different tissues focusing on how miRNA secretion drives the pathogenesis of metabolic disorders and whether fructose overconsumption is able to increase the risk of metabolic disease.

## Data Availability

The raw data supporting the conclusion of this article will be made available by the authors, without undue reservation and further inquiries can be directed to the corresponding author.
